# Continuous Monitoring During Atrial Lead Screw‐In, a Method to Reduce Atrial Lead Dislodgement

**DOI:** 10.1155/cric/4932942

**Published:** 2025-12-15

**Authors:** Binbin Luo, Longfu Jiang, Di Lu, Lu Zhang

**Affiliations:** ^1^ Department of Cardiology, Ningbo No. 2 Hospital, Ningbo, Zhejiang, China, nbws.gov.cn

**Keywords:** atrial screw-in lead, current of injury, dislodgement, real-time monitoring

## Abstract

The atrial lead is essential for atrial or dual‐chamber pacing, requiring stable fixation to ensure reliable electrical thresholds and sensing. Despite advancements in lead designs, atrial screw‐in leads still face a dislodgement rate of 0%–3.3%. This study introduces a novel technique for atrial lead fixation using real‐time injury current monitoring, offering a promising method to minimize lead dislodgement and enhance clinical outcomes.

## 1. Introduction

Pacemaker lead dislodgement is a prevalent complication, with incidence rates ranging from 0.5% to 4.0% [1–4]. Atrial lead dislodgement occurs more frequently than ventricular lead dislodgement [5]. Early reintervention can increase the risk of device‐related infections, deteriorate the doctor‐patient relationship, and escalate economic burdens. How to avoid dislocation during surgery becomes particularly important. Screw‐in electrodes induce localized trauma at the myocardial stimulation site, resulting in the generation of injury currents. By monitoring the presence and changes of the injury current, we can more effectively assess the fixation and safety of the electrode. We report the following three cases of atrial electrode implantation to further demonstrate.

## 2. Case Presentation

### 2.1. Case A

A 68‐year‐old female patient with third‐degree atrioventricular block underwent implantation of a left bundle branch pacing (LBBP) device. The ventricular lead used was a 3830 SelectSecure lead (Medtronic Inc.), while the atrial lead was a 5076 lead (Medtronic Inc.) positioned in the right atrium. The initial guidewire was replaced with a J‐shaped guidewire to facilitate precise electrode positioning. Under anteroposterior and left anterior oblique fluoroscopic guidance, the atrial lead was fixed to the right atrial appendage. The lead was then connected and clamped to the guidewire tail, and continuous monitoring was employed to observe intracavitary injury current changes during the insertion of the atrial appendage electrode.

**Figure 1:**
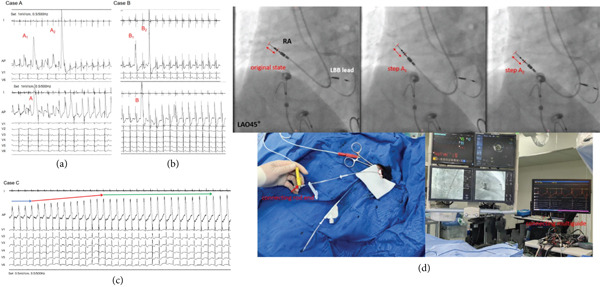
(a and b) Case A/B: Injury current was detected immediately upon electrode contact. After approximately 10 rotations, a significant increase in injury current was noted as the electrode head separated from the guidewire (A_1_, B_1_), while Point A_2_ (B_2_) is considered a secondary collision (refer to Step A_1_ and Step A_2_). Continued screwing resulted in no further increase in injury current. Similarly, injury current appeared upon electrode contact. After a jump in injury current during head separation (Point A/B), the atrial injury current gradually increased to a stable level as the electrode was screwed in. (c) Case C: With continuous monitoring demonstrated the appearance of injury current upon initial contact (blue arrow), with a progressive increase in injury current as the electrode was screwed in (red arrow), eventually reaching a stable level (green arrow). (d) The upper figure shows two different stages of the 5076 electrode during the screw‐in process, while the lower figure illustrates how to perform continuous screw‐in operations under continuous monitoring of injury current.

Upon contact of the electrode with the atrial tissue, an injury current is initially observed. As the screwing process continues (Figure 1d, LAO45° Step A_1_ to Step A_2_), there is a sudden and marked increase in the amplitude of the injury current, followed by a subsequent return to baseline levels (Figure 1a, Case A). Although the measured pacing threshold is 0.9 V, the sensing is 2.5 mV, and the impedance is 825 ohms, the lack of significant increase in injury during the screwing‐in process, along with the ease of dislodgement when gently pulling the lead, indicates insufficient fixation.

After repositioning the electrode, reestablish its position. Similarly, injury current appeared upon electrode contact. After a jump in injury current during head separation, the atrial injury current gradually increased to a stable level as the electrode was screwed in (Figure 1). Gentle pulling confirmed secure fixation, and no positional changes were observed when the patient was instructed to cough. The final measurements were a pacing threshold of 0.7 V, sensing of 2.7 mV, and impedance of 485 ohms (Video S1).

### 2.2. Case B

An 84‐year‐old female patient with atrial fibrillation and prolonged pauses underwent permanent pacemaker implantation. The left ventricle was measured at 3830 and the left atrium at 5076. Similar to Case A, the initial implantation of the atrial electrode did not cause significant atrial damage, and the sensing threshold was acceptable. However, slight traction resulted in dislodgement. The second implantation was stable. The pacing threshold was 0.5 V, sensing was 2.8 mV, and impedance was 763 ohms (Figure 1b, Case B).

### 2.3. Case C

An 81‐year‐old male patient with sick sinus syndrome underwent LBBP. The pacemaker model was A3DR01, utilizing dual 3830 leads. The 3830 lead was anchored intra‐atrial midseptum using C315 His sheath (Medtronic Inc.). Continuous monitoring at 2 V@0.5 ms using John Jiang′s connecting cable (Xinwell Medical Technology Co. Ltd., Ningbo, Zhejiang, China) demonstrated the appearance of injury current upon initial contact, with a progressive increase in injury current as the electrode was screwed in, eventually reaching a stable level (Figure 1c, Case C). When resistance was noticed, the implant stopped screwing in the leads and tested the pacing parameters. The final atrial measurements were a pacing threshold of 0.5 V, sensing of 3.2 mV, and impedance of 794 ohms.

## 3. Discussion

Due to the large number of transvenously implanted permanent pacemaker leads worldwide, lead dislodgement is a serious concern. While both active fixation electrodes and J‐shaped passive fixation electrodes have been used to prevent atrial lead dislodgement, this issue remains unresolved [1, 6, 7]. Atrial lead repositioning would have been straightforward at the very beginning, since there was no endocardial fibrous formation around the distal end of the lead or subclavian vein adhesion of the proximal segment of the lead [8, 9]. The introduction of active fixation electrodes offers the advantage of placing the lead anywhere in the atrium to achieve proper thresholds and amplitudes. However, cases of lead dislodgement cannot be predicted solely based on the threshold or sensing values at the time of implantation, as no differences have been found between dislodged and nondislodged leads in these parameters [10].

The appearance and continuous increase of intracavitary injury current serve as the basis for assessing penetration into myocardial tissue. Continuous monitoring during lead insertion not only allows for the observation of changes in intracavitary injury current but also helps assess the stability of the fixation and prevents electrode perforation. For the 5076 lead, the sudden surge in injury current during insertion is indicative of the helix penetrating and embedding into the myocardial tissue, followed by a gradual increase until stabilization. In contrast, both Case A and Case B exhibited two sudden increases in injury current without further subsequent increases, which may suggest that the lead did not properly engage with the tissue or became trapped in the pectinate muscles of the right atrial appendage. This could potentially lead to early displacement of the electrode. This hypothesis is further supported by the ease with which the lead can be withdrawn intraoperatively if it is not securely fixed. The 3830 lead, due to its nonprotruding design, shows a continuous increase in injury current until it is fixed when screwed into the atrial myocardium.

Importantly, while the present data do not yet provide direct proof that continuous injury current (COI) monitoring prevents long‐term lead dislodgement, our intraoperative experience consistently shows that, when no COI is observed during screw‐in, leads are more prone to dislodge. We suspect this occurs because the helix has not penetrated the atrial trabeculae. Thus, COI monitoring offers immediate confirmation of tissue engagement and serves as an intraoperative indicator of fixation security. At present, there is no established threshold for the magnitude of COI rise that guarantees stable fixation. We have therefore emphasized that defining quantitative thresholds for an “adequate” COI response will be a focus of future research.

Compared with the standard approach—screwing the lead in, connecting the cable, observing injury current afterward, and finally testing with traction—the COI‐guided method provides continuous, real‐time information throughout the screw‐in process. This allows operators to identify inadequate fixation before implantation is finalized and may reduce the need for repositioning. In other words, real‐time monitoring offers earlier and more reliable feedback than post hoc evaluation alone.

## 4. Limitation

The use of this continuous monitoring technique may further mitigate the risk of complications associated with pacemaker implantation. Importantly, we acknowledge that our present findings do not establish a definitive COI threshold for fixation and that larger studies are required to confirm long‐term outcomes.

## 5. Conclusion

By systematically performing intraoperative procedures under continuous monitoring, the adequacy of electrode implantation can be assessed. Continuous intraoperative monitoring of injury current during atrial lead screw‐in, coupled with an on‐the‐spot traction test, is a simple yet effective method to ensure proper lead fixation.

## 6. Take‐Home Message

These three cases highlight the importance of well‐controlled myocardial injury for maintaining electrode stability. Through real‐time monitoring of injury currents, the incidence of electrode dislodgement can be effectively reduced.

## Ethics Statement

The study was conducted in accordance with the Declaration of Helsinki. Ethical approval for reporting individual cases was waived by the Institutional Review Board of Ningbo No. 2 Hospital. Written informed consent was obtained from all patients (or their legal guardians) for publication of their clinical details, accompanying images, and supporting information (video). All patient information was anonymized to ensure confidentiality.

## Disclosure

The funders had no role in study design, data collection and analysis, decision to publish, or preparation of the manuscript.

## Conflicts of Interest

The authors declare no conflicts of interest.

## Funding

The original study was supported by the Medical Scientific Research Foundation of Zhejiang Province China (2022KY1137), the Research Foundation HwaMei Hospital, University of Chinese Academy of Sciences, China (2021HMKY02), and the Project of Ningbo Leading Medical & Health Discipline, China (2023Z191).

## Supporting information


**Supporting Information** Additional supporting information can be found online in the Supporting Information section. Video S1. This is a supporting information video of atrial lead screw‐in under continuous monitoring: 0:00–0:20 how to simultaneously monitor tissue damage and screw‐in during atrial lead connection. 0:21–0:31 as the atrial lead screw rotates, the screw extends twice, corresponding to two increases in injury current. 0:51–1:36 reposition the atrial lead and screw it in. With the lead secured, the atrial injury current increased progressively until stabilization, indicating electrode stability.

## Data Availability

All data underlying this case report are included within the article. No additional datasets were generated or analyzed. Therefore, no separate data files are available.
